# Analysis of tamoxifen and its metabolites in dried blood spot and volumetric absorptive microsampling: comparison and clinical application

**DOI:** 10.1016/j.heliyon.2021.e07275

**Published:** 2021-06-10

**Authors:** Baitha Palanggatan Maggadani, Yahdiana Harahap, Harmita, Samuel J. Haryono, Christoffel William Putra Untu

**Affiliations:** a Faculty of Pharmacy, Universitas Indonesia, Depok, 16424, Indonesia; b Indonesia Defense University, Bogor 16810, West Java, Indonesia; c Surgical Oncology Division, SJH Initiative-MRCCC Siloam Hospital, Jakarta, Indonesia

**Keywords:** DBS, VAMS, Recovery, Validation, Tamoxifen

## Abstract

This research was conducted to develop the Dried Blood Spot (DBS) and Volumetric Absorptive Microsampling (VAMS) method in the analysis of Tamoxifen (TAM) and its metabolites endoxifen (END), 4-hydroxytamoxifen (4-HT), and N-desmethyltamoxifen (NDT) using Ultra High Performance Liquid Chromatography-Tandem Mass Spectrometry (UPLC-MS/MS). This method was then applied to monitor TAM and its metabolites in breast cancer patients. The UPLC-MS/MS method was developed and validated with propranolol as the internal standard. The recovery and matrix effects on DBS and VAMS were investigated. The validation requirements were fulfilled by the methodology of analysis and sample preparation described in this study. Both VAMS and DBS extraction recoveries were satisfactory, with low variability. Extraction recovery in the VAMS sample was found to be slightly higher than in the DBS sample. Sample stability in DBS and VAMS was demonstrated for up to 2 months. Both of these methods were successfully applied for the analysis of TAM and metabolites in clinical patients. The mean concentrations obtained from the two methods were not significantly different.

## 1 Introduction

Tamoxifen (TAM) is a selective estrogen cancer receptor modulator (SERMs). TAM binds to estrogen receptors (ER), thus inhibits ER transcriptional activity and suppress tumor growth [1, 2]. TAM is widely used as a chemoprevention agent to reduce the risk of developing breast cancer [3, 4]. TAM is a prodrug and metabolized by CYP2D6 enzyme into more active metabolites such as endoxifen (END), 4-hydroxytamoxifen (4-HT), and N-desmethyltamoxifen (NDT). END contributes around 92% of TAM metabolism and has 30 to 100 times more activity than TAM and NDT [5, 6]. END plasma levels have been suggested to be strongly associated with the outcome of breast cancer treatment [7, 8]. Based on previous studies, the effectiveness of tamoxifen therapy in breast cancer patients depends on the concentration of END, where patients with END levels in serum above 5.9 ng/mL resulted in a lower risk of recurrence than patients with END level below this point [7].

TAM analysis has been done generally using plasma. Plasma is obtained by collecting venous blood using the venipuncture technique. However, this technique has disadvantages, such as invasive, requires large volumes of samples, gives patients a sense of discomfort, and inconvenient because liquid blood samples need to be frozen for transport [9]. Recently, microsampling techniques have been widely used for clinical purposes. This technique was done by collecting blood from the fingertips and spot the exact amount onto the paper media. Dried Blood Spot (DBS) and Volumetric Absorptive Microsampling (VAMS) are examples of the application of microsampling techniques. Both DBS and VAMS require less blood volume and a simple sampling procedure. The disadvantage of using DBS samples is that they are less sensitive, and the extraction time tends to be longer to obtain a high recovery. VAMS is the latest microsampling technique compared to DBS and has the advantage of being able to take samples consistently with a sampling capacity that varies from 10 to 30 μL [10, 11]. DBS PerkinElmer paper and VAMS tips Mitra® are different from their primary matrix component for absorbing the whole blood. The Mitra® tip is made of polymer, while DBS PerkinElmer paper is made of cotton [11]. This difference might affect their performance in the analysis of a compound. Therefore, a comparison of DBS and VAMS is needed to analyze TAM and its metabolites.

## 2 Material and methods

### 2.1 Reference standard samples and materials

TAM and NDT were purchased from Sigma-Aldrich (Singapore). Z-END and Z-4-HT were purchased from Santa Cruz Biotechnology (USA). Propranolol was purchased from Sigma-Aldrich (St. Louis, Missouri, USA). Formic acid, acetonitrile HPLC grade, and methanol for analysis were obtained from Merck (Darmstadt, Germany). PerkinElmer 226 papers were obtained from PerkinElmer (Waltham, Massachusetts, USA). VAMS Mitra® 20 μl was purchased from Neoteryx, USA. The whole blood (hematocrit (Hct%) value 34–43%) was obtained from the Indonesian Red Cross Society (Jakarta, Indonesia). The anticoagulant used is citrate phosphate dextrose adenine (CPDA1). Control blood was stored at refrigerator 4 °C and mix gently prior to use. Ultrapure water was used in this study and generated via Sartorius Water Filter system (Göttingen, Germany).

### 2.2 Preparation of solutions and standards

TAM, NDT, END, and 4-HT standards were diluted in methanol to obtain a 1,000 μg/ml stock solution concentration. Stock solutions for calibrator and quality control were prepared separately. The stock solution was diluted again with methanol to obtain intermediate solutions at 100 μg/ml of TAM and NDT and 10 μg/ml of END and 4HT. The working standard solution was prepared by pipetting an aliquot of the intermediate solution of all analytes into a volumetric flask and diluted with methanol to obtain the required concentration. Calibration and quality control samples were prepared by spiking the working solution in whole blood at a 1:20 ratio. Propranolol (PRO) as the internal standard stock solution was prepared in methanol at a concentration 10 μg/ml. Intermediate propranolol solutions were prepared by dilution of the stock with methanol to obtain a concentration of 1,000 ng/ml.

### 2.3 Sampel preparation

The sample preparation method in this study was referred to the previous study [12, 13]. DBS samples were prepared by pipetting 20 μl of the spiked blood onto the DBS paper and dried for 1–3 h. The whole spot was cut out. The disc was cut in half and transferred into a polypropylene microtube 1.5 ml. The VAMS samples were prepared by dipping the Mitra® tip in the spiked whole blood until the tip was colored with blood; let it dip for 2 s. The tip was dried at room temperature for 1–3 h. After the tip was completely dried, the tip was removed from the handle and put into microtubes. Both samples (DBS and VAMS) were then extracted using the same method and condition.

Extraction of analytes by protein precipitation method using methanol assisted by ultrasonication. 1000 μl methanol containing 100 ng/ml PRO were added to the microtubes. Then the sample cup was vortexed for 1 min and sonicated for 15, 25, and 45 min. 850 μl of the sample mixture was dried under nitrogen (50 °C) for 15 min, and the dried extract was reconstituted in 100 μl 0.1% formic acid – 0.1% formic acid in acetonitrile (5:95). Centrifugation of the sample mixture was done for 10 min at 805 x g. 70 μl of the supernatant was pipetted and transferred to an autosampler vial, 10 μl was injected into the LC-MS/MS system.

### 2.4 LC-MS/MS

LC-MS/MS system using Waters Xevo TQD Triple Quadrupole with MassLynx Software controller (Waters, Milford, USA). Reversed-phase Chromatographic analysis using Acquity UPLC C18 BEH column (2.1 × 100 mm; 1.7 μm). Mobile phase A consisted of 0.1% water and mobile phase B 0.1% formic acid in acetonitrile. Elution system using gradient at a flow rate of 0.2 ml/min with total analysis time was 5 min. The initial composition of eluent was A: B (5:95), which was hold for 3 min. The ratio was changed to 70:30 and held for 2 min.

Mass detection performed in Triple Quadrupole (TQD) mass analyzer in Multiple Reaction Monitoring (MRM) analysis modes with an electrospray ionization source positive mode.

The mass parameter setting was as follows: The capillary voltage +3500 V; Source gas using Nitrogen, temperature 450 °C, and the flow rate at 700 L/h. Argon was used as the collision gas. Collision energies were 27, 30, 27, 24, and 20 eV for TAM, END, 4-HT, NDT, and IS, respectively. Transition from the precursor into a product was set at m/z 372.28 > 72.22 for TAM; 374.29 > 58.22 for END; 388.29 > 72.19 for 4-HT; [bm1] 358.22 > 58.09 for NDT; and 260.20 > 116.20 for propranolol.

### 2.5 Validation assay

Validation assay was performed using DBS PerkinElmer 226 and VAMS Mitra® sample based on Food and Drug Administration (2018) and European Medicines Agency (2011) guidelines for validation of bioanalysis [14, 15].

### 2.6 LLOQ and calibration curve

Calibration samples were prepared by spiking the working solution in whole blood at a 1:20 ratio. 50 μl working solutions were spiked into 950 μl blank blood with to obtain eight calibration levels of samples containing TAM (2.5–200 ng/ml), NDT (2–600 ng/ml), END (2.4–40 ng/ml), and 4-HT (1.5–30 ng/ml). Blank, zero, and calibration samples were spotted to PerkinElmer 226 paper and VAMS. Samples were extracted using the optimum method and analyzed within 24 h. Replicates at each concentration have been analyzed as mentioned above. Calibration curves were generated by plotting the area ratio (PAR) of the analyte peak and the IS peak versus the nominal concentration of the calibration sample. The acceptance condition for the LLOQ was within 20 % of CV value and %bias, while the other concentrations were 15 %.

### 2.7 Selectivity

Selectivity was determined by preparing six batches of LLOQ samples in 6 different sources of whole blood (Hct 0.34–0.43). DBS and VAMS sample was prepared according to the optimum method. The possibility of interferences was observed in the blank matrix at the retention time of each analyte.

### 2.8 Accuracy and precision

The accuracy and precision of the analytical method were assessed by comparing the LLOQ (Lower Limit of Quantitation), LQC (Low Quality Control), MQC (Middle Quality Control) and HQC (High Quality Control) analyze on intra and inter-assays. Both assays were performed with five replicates. Inter-assays are carried out in 3 different runs for a minimum of 2 days. QC samples were made by spiking QC working solution into blank blood to obtain LLOQ, LQC, MQC, and HQC concentration. The LLOQ and QC sample concentrations for TAM, END, 4HT and NDT were 2.5; 2.5; 1.5 and 2 ng/ml, respectively (LLOQ), 7.5; 7.5; 4.5; and 6 (LQC), 60; 18; 15 and 250 ng/ml, respectively (MQC), 150; 35; 25 and 500 ng/ml, respectively (LQC). LLOQ and QC samples were applied onto PerkinElmer 226 paper and VAMS. Samples were extracted using optimum method and analyzed within 24 h Accuracy and precision were acceptable if % CV and bias of LLOQ were below 20% while other concentrations were below 15%.

### 2.9 Carry over

The effect of the previous injection was investigated by injecting a DBS and VAMS blank after injection of the analyte with the highest concentration on calibration. The carry-over test meets the requirements if the blank sample area does not exceed 20% LLOQ in the analyte and 5% of the analyte in the standard.

### 2.10 Recovery

Two kinds of sample solutions were prepared: (A) The extracted samples of LQC, MQC, and HQC were prepared by spiking the analyte into a blank matrix before the extraction, as mentioned in sample preparation. (B) The unextracted samples of LQC, MQC, and HQC were prepared by spiking the analyte after the matrix extraction to represent 100% recovery. Both assays were performed with three replicates for each concentration. Recovery is calculated from the ratio A/B∗100%. Recovery experiments were performed for both DBS and VAMS.

### 2.11 Matrix effect

The effect matrix is assessed by diluting the QC working solution to obtain LQC and HQC concentration and spiking to whole blood. For this assay, two kinds of solutions were prepared: (B) the analytes were spiked after blank matrix extraction. (C) Neat solution of standards in the mobile phase. Matrix effect or absolute recovery is calculated from the ratio B/C ∗ 100%. IS normalized matrix factor is measured by comparing the matrix factor analyte and IS. The acceptance criteria for the matrix effect is a maximum of 15% CV. Matrix effect experiments were performed for both DBS and VAMS.

### 2.12 Stability

The stability of the standard solution was tested on methanol solutions of TAM, END, NDT, 4HT. This experiment aimed to evaluate the stability of the standard solution in storage for 24 h at room temperature during preparation and at storage conditions of -20 °C for up to 60 days.

Stability of DBS and VAMS samples were evaluated for whole blood stability, 24 h (benchtop and autosampler stability), and long term at room temperature storage. Benchtop stability was evaluated every 6 h until 24 h to estimate the stability of the sample during processing. Benchtop and long-term stability were evaluated using LQC and HQC samples at room temperature storage. DBS samples were stored in a plastic sealed bag with desiccant, while VAMS samples were stored in VAMS clamshell and put in a plastic sealed bag with desiccant. Both were protected from direct sunlight. In addition, the stability of samples was evaluated in low (-20 °C) and high temperatures (40 °C) for 24 h. This covers conditions that may be encountered during the transport of the samples to the point of analysis. % bias and CV should not exceed 15%

## 3 Results and discussion

### 3.1 Sample preparation

**Figure 1:**
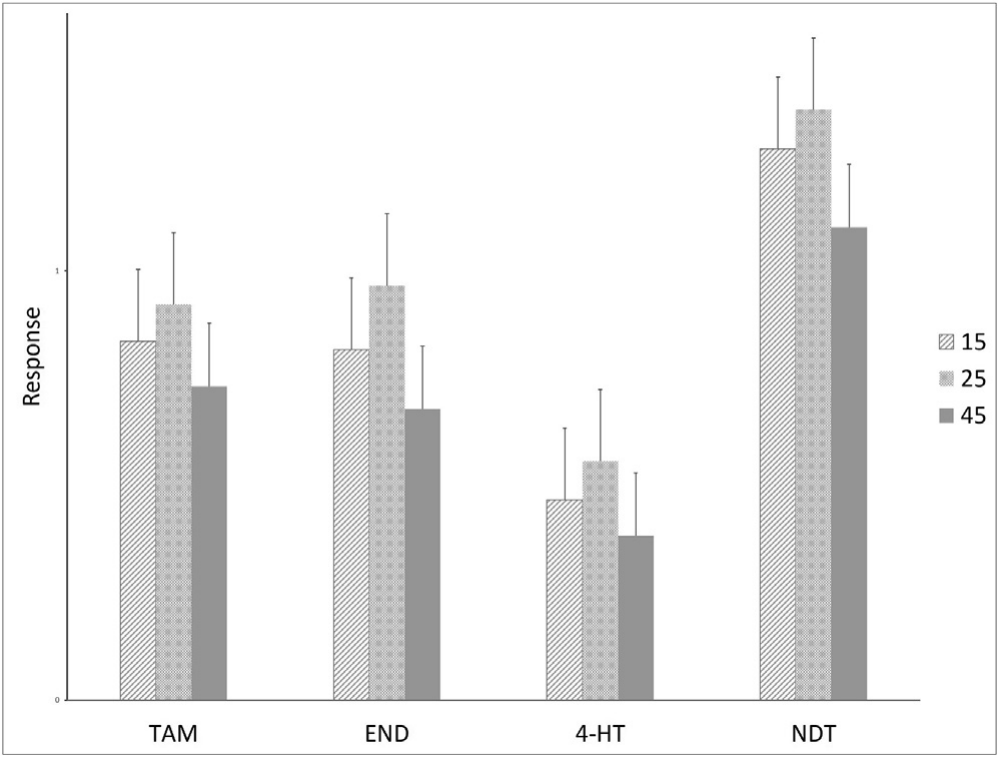
TAM, END, 4HT and NDT response to IS by sonication for 15, 25 and 45 min.

The extraction process was optimized by varying the drying and sonication time parameters. The drying of the VAMS and DBS tips were varied at 60, 120, and 180 min. Extract samples from VAMS and DBS that were dried for 120 min yielded a higher response than 60 min. Meanwhile, increasing the time to 180 min did not show any significant results. Sonication was aimed to improve the extraction of analytes from the biological matrix. Sonication has been shown to enhance recovery in several studies [9, 16, 17, 18]. The sonication time was optimized at 15, 25, and 45 min Figure 1 illustrates the response (Peak Area Ratio) of each metabolite to IS after sonication for 15, 25, and 45 min. As shown in Figure 1. Sonication of 25 min resulted in a higher response compared to 15 min while increasing the duration to 45 min caused a decrease in response which was probably caused by damage to the analyte due to the heat generated from the sonication process.

### 3.2 Chromatography condition

**Figure 2:**
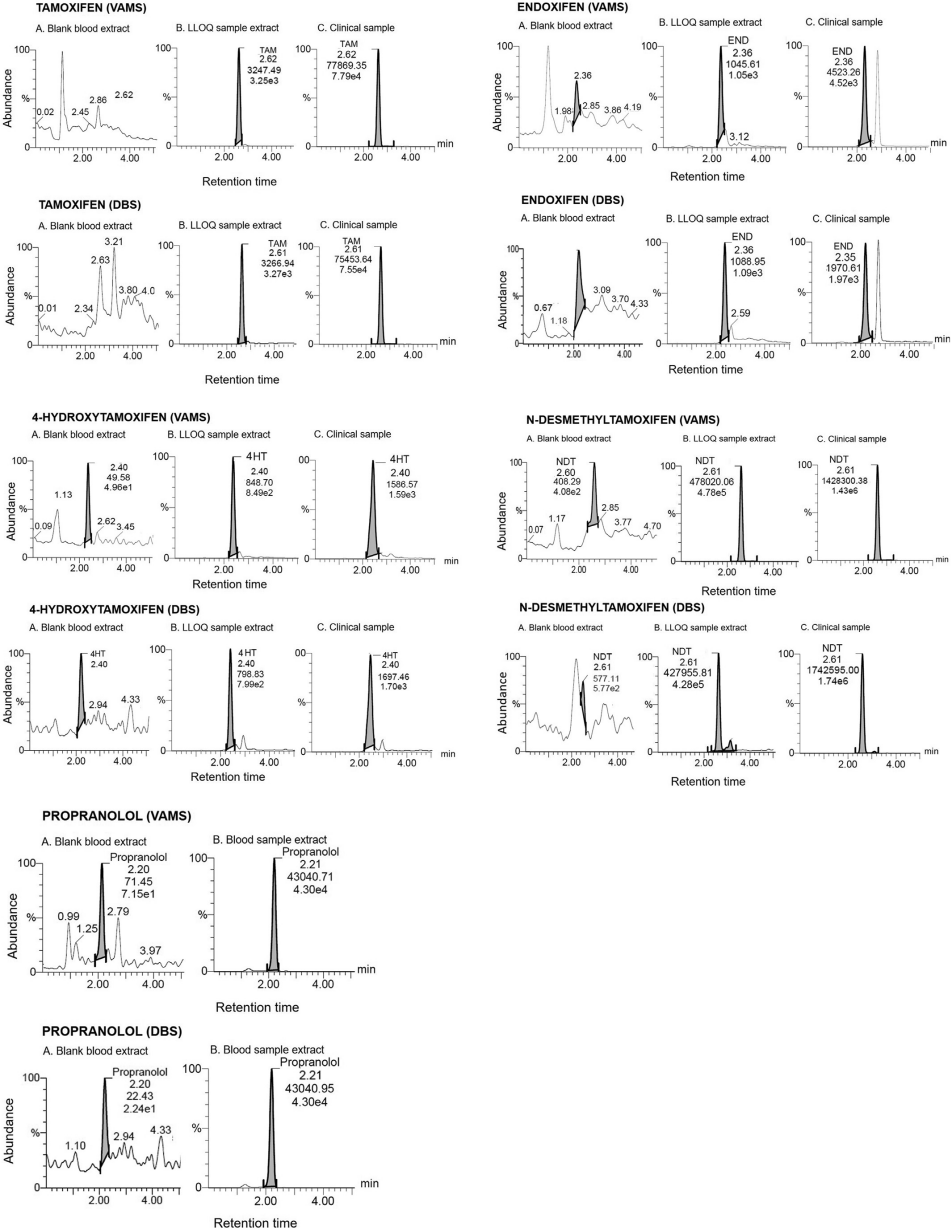
Chromatograms obtained from DBS and VAMS: (A) Blank blood extract per channel (B) LLOQ spiked sample extract and (C) Clinical sample extract.

The LC-MS/MS method has proven to be an effective system for the determination of TAM and its key metabolites in DBS samples. Optimum separation conditions have been obtained by modifying multiple parameters, such as mobile phase composition and mixture, flow rate, and gradient profile. An Acquity UPLC C18 BEH column (2.1 × 100 mm; 1.7 μm) was used in this study. A linear gradient separation was used with 5–95% of mobile phase A-B from 0 to 3 min, then adding gradient 70–30% of mobile phase B, which was held for 2 min. These systems successfully separated analytes within 5 min Figure 2 shows the chromatograms of blank blood, spiked blood (LLOQ), and the clinical samples of TAM and metabolites derived from DBS and VAMS. The retention time of TAM, END, 4HT, NDT QC sample, and patient VAMS and DBS samples were 2.61–2.62; 2.35–2.36 2.40; and 2.61 min, consecutively. Retention times of IS were 2.21 min.

### 3.3 Validation assay

Full validation in DBS and VAMS was conducted in this study. The Calibration model was created using two sets of eight standard concentration levels were prepared and analyzed in three different batches. Linear responses were achieved for TAM, END, NDT, 4HT ng/ml with a correlation coefficient of ≥0.9926. As shown in Tables 1 and 2, the intra and inter-assay precision and accuracy experiments performed on LLOQ and QC samples (LQC, MQC, and HQC) were fulfilled the requirements of EMA and FDA. Intra and inter-assay imprecision in DBS was in the range of 2.24–13.07% (15.85% for LLOQ) and 5.01–12.03%, respectively. Intra and inter-assay imprecision in VAMS were in the range of 0.58–11.18% and -12.64 to 15.35%, respectively. Accuracy intra and inter-assay in DBS was estimated at %bias -17.29(LLOQ) to 14.00% and -19.69 (LLOQ) to 14.49%, respectively. Accuracy intra and inter-assay in VAMS was estimated at %bias 3.50–9.48% and -1.02 to 5.79%, respectively.

**Table 1:** Summary of validation in DBS.

Analyte	Linear regression weighing factor 1/x	Quality control	Nominal Concentration (ng/ml)	Precision (%CV)		Accuracy (%Bias)	
				Intra-assay	Inter-assay	Intra-assay	Inter-assay
TAM	y = 0.0148x+0.0355 r = 0.9987	LLOQ	2.50	15.85	12.03	-17.29	-19.69
		LQC	7.50	8.90	8.05	14.00	-2.96
		MQC	60.00	5.45	6.30	6.97	7.14
		HQC	150.00	2.24	7.31	-8.82	12.33
NDT	y = 0.0138x+0.0426 r = 0.09967	LLOQ	2.00	5.54	8.96	9.67	14.49
		LQC	6.00	7.17	6.54	-11.22	-11.22
		MQC	250.00	11.21	9.71	-14.81	13.82
		HQC	500.00	4.07	9.18	-10.72	-1.34
END	y = 0.0204x-0.0294 r = 0.09965	LLOQ	2.50	13.07	7.94	-12.48	16.71
		LQC	7.50	7.65	5.67	6.15	6.15
		MQC	18.00	8.80	7.89	11.51	11.16
		HQC	35.00	4.62	7.11	-10.28	10.28
4-HT	y = 0.0277x-0.0131 r = 0.09926	LLOQ	1.50	8.46	10.20	12.08	18.90
		LQC	4.50	6.42	5.01	-12.89	-9.29
		MQC	15.00	7.50	6.89	9.09	9.09
		HQC	25.00	7.24	8.09	-4.78	-8.82

Abbreviation LLOQ: Lower limit of Quantification, LQC: low level, MQC: medium level, HQC: high level, CV: variability, number of experiments (n): 5.

**Table 2:** Summary of validation in VAMS.

Analyte	Linear regression weighing factor 1/x	QC	Conc. (ng/ml)	Precision (%CV)		Accuracy (%Bias)	
				Intra-assay	Inter-assay	Intra-assay	Inter-assay
TAM	y = 0.0363x+0.1765 r = 0.9985	LLOQ	2.50	8.05	11.61	5.51	2.23
		LQC	7.50	2.64	10.47	6.03	-1.02
		MQC	60.00	4.31	9.48	3.73	1.97
		HQC	150.00	5.46	-7.24	4.52	1.98
NDT	y = 0.1272x+2.8675 r = 0.9992	LLOQ	2.00	10.03	15.35	9.48	1.29
		LQC	6.00	6.34	6.16	5.85	2.21
		MQC	250.00	0.58	4.84	3.91	1.82
		HQC	500.00	3.35	5.30	3.84	-0.16
END	y = 0.0083x+0.0164 r = 0.9974	LLOQ	2.50	5.19	13.91	7.35	-0.07
		LQC	7.50	6.48	12.12	7.25	0.52
		MQC	18.00	8.94	4.06	3.50	0.44
		HQC	35.00	1.99	8.65	3.91	5.79
4-HT	y = 0.0083x+0.0669 r = 0.9902	LLOQ	1.50	9.12	-14.94	6.18	1.22
		LQC	4.50	11.18	-12.64	5.13	2.3
		MQC	15.00	4.89	-8.16	6.33	3.58
		HQC	25.00	4.59	2.90	6.97	2.41

Abbreviation LLOQ: Lower limit of Quantification, LQC: low level, MQC: medium level, HQC: high level, CV: variability, number of experiments (n): 5.

The selectivity test results revealed that neither interferences were observed above 20% of the LLOQ area and 5% of the IS. The maximum carry-over of TAM, END, 4HT and NDT of DBS and VAMS samples were 6.09% and 7.11%; 16.83% and 3.21%; 12.55% and 1.98%; 12.09% and 3.67% respectively. The carry-over for IS was below 0.65%. Those values met the acceptance, not to exceed 20% for LLOQ and 5% for IS.

The stability of all metabolites in methanol showed %bias below 6% when stored at room temperature with an air conditioner (20–25 °C) for 24 h and in the freezer (-20 °C) for one month. The solution was kept in an amber glass vial and protected from light. All metabolites also showed good stability under processing and autosampler with the % bias and % CV values below 15%. TAM, END, 4-HT, and NDT in VAMS and DBS samples were stable during room temperature storage (air-conditioned 20–25 °C) period for up to 1 month. The stability of DBS and VAMS samples was also tested after storage at -20 °C and 40 °C for 24 h. This temperature was chosen to mimic possible conditions during sample transport to the point of analysis. %CV and bias were under 15% for all analytes in both DBS and VAMS indicates all analytes were stable under storage at tested temperature for 24 h. However, a decrease in all metabolites was observed after storage at high temperatures. This should be taken into consideration that if the shipment of the sample is more than 24 h, it is recommended that it be stored at a temperature not exceeding 25 °C. The stability data of all metabolites in DBS and VAMS were presented in Table 3.

**Table 3:** Stability data of TAM, NDT, END, and 4-HT in DBS and VAMS.

Compound	TAM		END		4HT		NDT	
	%CV	%bias	%CV	%bias	%CV	%bias	%CV	%bias
**Stock solution in methanol (20-25**^**0**^**C)**								
Short term (24h)	0.34	-0.86	0.31	-1.36	0.20	-1.37	0.29	0.56
Long term (60 d)	0.02	-4.40	0.46	-5.66	1.00	-2.59	0.60	-3.98
**In VAMS sample (LQC;HQC)**								
Autosampler (24h)	3.66 4.11	-3.60 3.72	2.39 1.17	-4.86 3.8	7.42 3.69	1.76 0.84	2.82 2.72	-6.26–1.59
Benchtop (24h, 20–25 °C)	6.64 3.72	-6.01–1.99	4.69 5.39	-8.87–2.23	8.68 7.55	2.31–3.38	4.17 5.25	03.78–0.11
Long term (20–25 °C) (60 d)	1.45 0.45	-10.75–3.44	1.92 0.97	-7.70–5.55	0.90 0.29	-12.75–10.89	2.66 0.22	-4.35 1.55
During transport (LQC; HQC)								
-20 °C	2.98 3.27	-2.27 5.31	8.23 4.03	-4.71–3.49	1.99 3.69	-4.83–3.38	6.54 7.05	-2.73–4.76
40 °C	5.71 10.61	-10.82–13.28	8.36 6.47	-12.86;-10.90	10.10 5.12	-13.48–10.03	2.02 1.69	-11.98–11.09
**In DBS sample (LQC;HQC)**								
Autosampler (24h)	1.92 0.97	-3.60 3.72	0.90 0.29	-4.86 4.40	0.87 0.61	-4.03 1.84	1.45 0.42	-6.26–1.59
Benchtop (24h, 20–25 °C)	5.51 5.04	-7.05–4.54	1.93 2.60	-11.16–12.07	7.41 1.63	-4.23–10.44	3.77 0.61	-14.01–12.35
Long term (20–25 °C) (60 d)	4.92 3.66	-11.33–10.31	3.66 3.63	-10.31–12.98	8.72 3.03	-6.94–10.36	2.02 0.13	-5.37–2.48
During transport (LQC; HQC)								
-20 °C	3.66 4.11	-2.73–1.99	2.39 1.17	-4.41 2.23	2.64 3.69	-1.96 2.14	2.82 2.72	7.78 5.47
40 °C	6.67 12.02	-11.35–11.12	12.53 9.16	-13.51–11.62	11.41 8.70	-12.97–10.31	13.02 7.92	-13.64–9.38

Abbreviation LQC: low level, HQC: high level, CV: variability, number of experiments (n): 3.

### 3.4 Recovery & matrix effect using DBS and VAMS

The recovery test was performed using three replica samples at three concentrations of the extracted sample (A) and unextracted sample (B). The extracted sample was prepared by spiking the analyte into the blood matrix. The sample was spotted on the DBS card and extracted as described in the section on sample preparation. Unextracted samples were obtained by spiking the analyte after DBS extraction from the blank matrix. Recovery for extraction was measured as the response of the detector (Peak Area of Analytes) (A/B)∗100%. The FDA and EBF state that analyte recoveries do not have to be 100%, but analyte recovery and internal standards must be consistent, precise, and reproducible [15, 19].

The average TAM and NDT recovery in VAMS were above 90%, whereas END and 4-HT showed lower recovery, 79.70%–88.63% (Table 4). When the same method was applied to DBS, it showed lower data recovery for almost all analytes. Recovery on DBS ranged from 75.12% (LQC 4HT) to 89.03 (HQC 4HT). The highest average recovery is shown in NDT, while the average recovery shown by TAM, END, and 4HT was in the range of 84%. Recovery produced by VAMS tends to be better than the recovery produced by DBS PerkinElmer 226 in the analysis of TAM, END, 4-HT, and NDT. This difference might be influenced by the type of matrix material of the biosampling device. VAMS has a base material made from a polymer with hydrophilic properties; meanwhile, DBS PerkinElmer 226 is made out of cotton, which has hydrophobic properties [20]. %CV value from recovery data of all analytes showed no significant variability, indicating the consistency and precision of extraction recovery. The % recovery also tended to be concentration-independent. Extraction recovery is a valuable tool for determining extraction performance. Several experiments on dried blood samples reported reduced recovery values at higher Hct, lower doses, and samples that had been dried for a few weeks, resulting in a bias for the calculated concentration [21]. This decrease in recovery is probably due to the strong hydrogen bonds formed between the analyte and the cellulose matrix in the DBS paper [22]. Antunes et al. indicated that the impact of Hct increased the percent bias in Hct% above 40%. Analyte concentrations must be corrected in patients with a Hct% greater than 40% to achieve the correct value [17]. The effect of Hct% will be the focus of our following research so that this method can be applied further for therapeutic drug monitoring.

**Table 4:** Recovery and matrix effect of TAM, NDT, END, and 4-HT.

Analyte	Biosampling	Quality control	Recovery ± CV (%)	Matrix factor (absolute Recovery ± CV(%))	Internal Standard-normalized Matrix factor ±%CV
TAM	DBS	LQC	86.84 ± 5.09	81.80 ± 5.23	0.89 ± 5.33
		MQC	82.12 ± 0.85		
		HQC	84.30 ± 6.42	85.88 ± 1.78	0.94 ± 3.81
	VAMS	LQC	91.13 ± 1.37	96.77 ± 2.04	0.98 ± 3.72
		MQC	92.02 ± 0.94		
		HQC	92.54 ± 1.56	96.34 ± 2.20	0.98 ± 1.18
NDT	DBS	LQC	80.41 ± 1.99	83.90 ± 3.57	0.92 ± 5.73
		MQC	91.86 ± 0.70		
		HQC	87,31 ± 4.57	87.31 ± 2.66	0.95 ± 5.30
	VAMS	LQC	91.72 ± 0.85	95.06 ± 1.82	0.96 ± 3.10
		MQC	91.92 ± 1.17		
		HQC	91.77 ± 0.73	95.83 ± 0.75	0.97 ± 2.09
END	DBS	LQC	86.53 ± 3.90	78.71 ± 1.52	0.96 ± 2.05
		MQC	89.21 ± 4.03		
		HQC	78.59 ± 3.62	80.21 ± 3.97	0.96 ± 4.67
	VAMS	LQC	88.63 ± 0.89	96.86 ± 0.74	0.98 ± 2.36
		MQC	87.12 ± 1.07		
		HQC	87.24 ± 1.60	95.70 ± 1.65	0.97 ± 2.87
4-HT	DBS	LQC	75.12 ± 5.27	85.02 ± 4.56	0.93 ± 6.54
		MQC	88.32 ± 9.00		
		HQC	89.03 ± 3.48	81.46 ± 2.41	0.89 ± 3.28
	VAMS	LQC	80.48 ± 2.12	95.54 ± 2.17	0.97 ± 4.36
		MQC	79.70 ± 1.57		
		HQC	88.27 ± 1.64	96.27 ± 1.23	0.98 ± 2.99
PRO	DBS		81.70 ± 0.96	91.76 ± 3.57	
	VAMS		91.75 ± 0.50	97.92 ± 2.11	

Abbreviation LQC: low level, MQC: medium level, HQC: high level, CV: coefficient of variation n = 3.

The matrix effect (ME) was observed from the percent ion suppression or enhancement in the area produced by the unextracted sample (B) compared to the area of sample C. The result showed ionization suppression below 5% in all analytes extracted from VAMS. Ion suppression was greater in all analytes extracted from DBS, which was in the range of approximately 20%. END and 4HT demonstrated to be more affected by matrix effect than TAM and NDT. This was probably related to the levels of END and 4HT in the blood, which were much lower than TAM and NDT. However, IS normalized matrix factor for VAMS and DBS was between 0.89 and 0.98, suggesting that the matrix effect is identical for both analytes and IS. This implied that the IS used in this study was adequate to minimalize the analysis errors caused by ME [23].

### 3.5 Application of the method

This method was applied to 30 breast cancer patients who had received tamoxifen 20 mg daily for at least 8 weeks. Table 5 shows the demographics of the patients. The study was carried out in accordance with the Helsinki Declaration and was authorized by the Ethics Committee of the MRCCC Siloam Semanggi Jakarta. Each participant had provided informed consent and had been informed about the analyses and procedures. The median age of the patients was 46.81, with a median BMI of 22.57. All patients were from Indonesia, with the majority Javanese. Blood was drawn from the fingertips, and 20 uL was collected directly using VAMS, while for DBS, an aliquot of blood was collected in an EDTA microtube, and 20 μl of blood was pipetted and spotted on DBS paper. The VAMS and DBS samples were dried at room temperature for 2 h and extracted with methanol. The developed sample preparation and analysis methods were used for further analysis.

**Table 5:** Patients demographic characteristic.

Characteristic	N = 30
Age (years)	
Median	46.81
Range	39–67
BMI	
Median	22.57
Range	18.95–27.22
Race/ethnicity	
Javanese	12
Chinese	9
East Indonesia	3
Batak/Minang	3
other	3
Menopause Status	
Pre-menopause	20
Post-Menopause	10
Tamoxifen duration (months)	
Median	20
Range	2–48
CYP2D6 inhibitor drugs	None

As shown in Table 6, The mean TAM, END, 4HT and NDT concentrations in VAMS and DBS were 98.09 vs. 86.96; 11.17 vs. 10.67; 3.53 vs. 3.25; 220.66 vs. 206.79 ng/ml, respectively. Both samples were analyzed using the same sample preparation and analysis method. DBS and VAMS concentrations of TAM, END, 4HT, and NDT were all within the validated range of the assay. Among metabolites, NDT showed the highest concentration, while 4HT was the lowest. END and 4HT are important metabolites since they have the highest affinity for estrogen receptors. The mean END showed a higher concentration value of 3.2 fold compared to 4HT indicates the domination of END over 4HT. similar studies were reported previously by Antunes, et al with a 5.7 and 6.5 -fold difference between END and 4HT [17, 24]. As shown in Table 6, the concentrations of metabolites in VAMS and DBS were very variable, with the difference between highest and lowest were 6.45 vs. 5.95; 7.94 vs. 8.03; 4.23 vs. 4.92; and 7.89 vs. 5.39 fold for TAM, END, 4-HT, and NDT, respectively. END showed the highest variability among other polymorphisms of metabolizing enzymes like CYP2D6 and CYP 3A4, and drug interactions may contribute to interindividual pharmacokinetic variability [7, 25, 26].

**Table 6:** Summary of analysis of TAM and metabolites in patients.

Analytes	Blood sampling	Mean ± SD	Concentration in ng/ml	
			Range	P value
TAM	VAMS	98.09 ± 52.18	36.14–233.31	0.17
	DBS	86.96 ± 39.24	31.89–189.80	
END	VAMS	11.17 ± 5.37	3.78–30.03	0.35
	DBS	10.67 ± 5.20	3.86–31.01	
4HT	VAMS	3.53 ± 1.93	1.55–6.56	0.23
	DBS	3.25 ± 1.36	1.57–7.72	
NDT	VAMS	220.66 ± 70.99	48.80–385.01	0.24
	DBS	206.79 ± 79.10	53.94–291.16	

The mean concentration of all analytes extracted from VAMS showed a slightly higher value than in DBS. An unpaired t-test was conducted on DBS and VAMS to determine whether there was a significant difference in concentration level between the two groups. SPSS version 22.0 was used for the analysis. The p-values for all analytes in DBA and VAMS were more than 0.05, suggesting that there is no statistically significant difference between the two biosampling methods.

**Figure 3:**
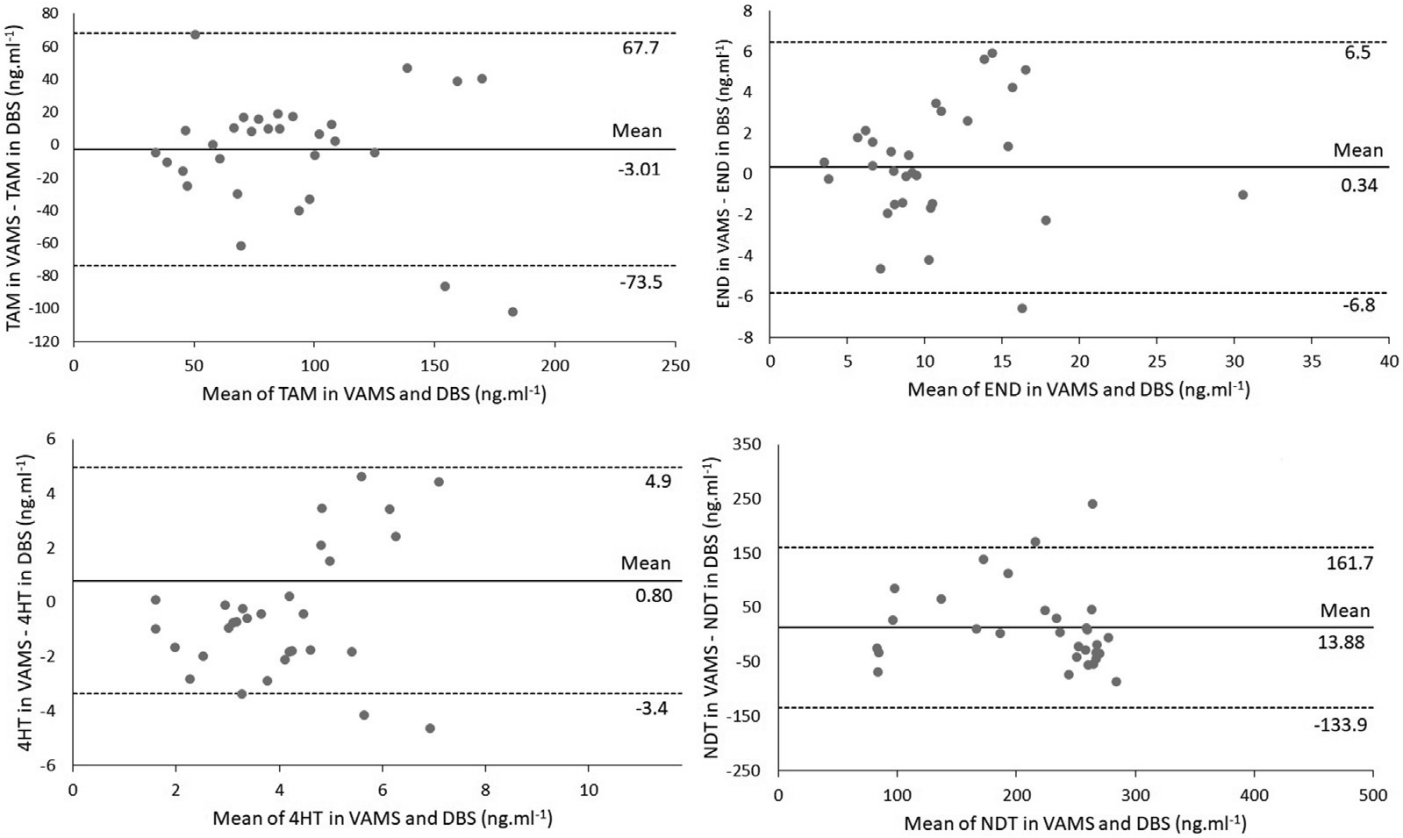
Bland Altman concentration plot TAM, END, 4HT and NDT in DBS vs. VAMS in clinical samples (N = 30).

Mean concentration of TAM and metabolites in VAMS and DBS were compared using Bland Altman Plots (Figure 3). Statistical analysis was done using SPSS version 22.0. Mean difference of TAM in VAMS and DBS was -3.01; END 0.34; 4HT 0.8 and NDT 13.88. Of 30 data, two values were out of ±1.96 deviation area for TAM, while END only one value, 4HT 3 value, and NDT 2 values. Differences were randomly scattered around the mean, suggesting no statistical error relative to the TAM and metabolite ratios.

The END values showed a gap between the lowest and highest levels, i.e., 10-fold. This significant difference is likely due to the variability of the pharmacokinetic profile of each individual or the concomitant drug that inhibits TAM metabolism. CYP2D6 and CYP3A4 play an essential role in the metabolism of tamoxifen to END. Polymorphisms of these enzyme-coding genes, particularly CYP2D6, have been shown to cause variability in levels and outcomes in several studies [27, 28, 29, 30]. The serum level of 5.9 ng/ml was the threshold for effectiveness of TAM therapy. Antunes and Jager converted these values in DBS to 3.3 and 3.9 ng/ml [17, 31]. Of the 30 patients analyzed, 2 had END levels in whole blood less than 3.9 ng/ml. The cause of the low levels of these two patients needs to be further studied to ensure the effectiveness of TAM therapy. According to the results of this study, VAMS as well as DBS may be used to examine TAM levels and metabolites in clinical patients. However, it must be demonstrated that this peripheral blood sampling method is interchangeable with the standard TDM sampling method (plasma or serum). The interaction of drugs with plasma components and accumulation in red blood cells might result in a concentration difference between plasma (or serum) and whole blood. Furthermore, due to the drug distribution process, the concentration in the peripheral blood may differ from the concentration in the venous [22]. Further research is needed to evaluate the blood vs. plasma relationship and the venous vs. peripheral blood correlation to address this issue.

## 4 Conclusion

The method developed for the quantification of TAM, END, 4HT and NDT in DBS PerkinElmer 226 was successfully validated according to FDA and EMA guidelines. Extraction and absolute recovery in the VAMS sample were shown to be slightly higher than in DBS. Both methods can be applied for the examination of TAM levels and metabolites in clinical patients. The mean concentration of analysis derived from both methods did not indicate a significant difference. The analytes in DBS and VAMS also showed stability in storage at room temperature for up to two months. Both VAMS and DBS were suitable for use in clinical trials and therapeutic drug monitoring. On examination of 30 patients in the study, two patients showed low END levels. Further investigations need to be carried out to ensure every patient has a successful TAM therapy.

## Declarations

### Author contribution statement

Baitha Palanggatan Maggadani: Performed the experiments; Analyzed and interpreted the data; Wrote the paper.

Yahdiana Harahap: Conceived and designed the experiments; Contributed reagents, materials, analysis tools or data; Wrote the paper.

Harmita: Analyzed and interpreted the data; Wrote the paper.

Samuel J Haryono: Conceived and designed the experiments; Contributed reagents, materials, analysis tools or data.

Christoffel William Putra Untu: Performed the experiments; Wrote the paper.

### Funding statement

This work was supported by 10.13039/501100006378Universitas Indonesia.

### Data availability statement

Data will be made available on request.

### Declaration of interests statement

The authors declare no conflict of interest.

### Additional information

No additional information is available for this paper.
